# Health Behaviors of Austrian Secondary School Teachers and Principals at a Glance: First Results of the *From Science 2 School* Study Focusing on Sports Linked to Mixed, Vegetarian, and Vegan Diets †

**DOI:** 10.3390/nu14051065

**Published:** 2022-03-03

**Authors:** Katharina C. Wirnitzer, Clemens Drenowatz, Armando Cocca, Derrick R. Tanous, Mohamad Motevalli, Gerold Wirnitzer, Manuel Schätzer, Gerhard Ruedl, Werner Kirschner

**Affiliations:** 1 Department of Subject Didactics and Educational Research and Development, University College of Teacher Education Tyrol, 6010 Innsbruck, Austria; derrick.tanous@student.uibk.ac.at (D.R.T.); seyed.motevalli-anbarani@student.uibk.ac.at (M.M.); 2 Department of Sport Science, Leopold-Franzens University of Innsbruck, 6020 Innsbruck, Austria; armando.cocca@uibk.ac.at (A.C.); gerhard.ruedl@uibk.ac.at (G.R.); werner.kirschner@uibk.ac.at (W.K.); 3 Research Center Medical Humanities, Leopold-Franzens University of Innsbruck, 6020 Innsbruck, Austria; 4 Division of Sport, Physical Activity and Health, University of Teacher Education Upper Austria, 4020 Linz, Austria; clemens.drenowatz@ph-ooe.at; 5 AdventureV & Change2v, 6135 Stans, Austria; gerold@wirnitzer.at; 6 Special Institute for Preventive Cardiology and Nutrition—SIPCAN, 5061 Elsbethen, Austria; m.schaetzer@sipcan.at

**Keywords:** health, body weight, physical activity, exercise, plant-based, nutrition, fruit, vegetables, lifestyle

## Abstract

Lifestyle behaviors are key contributors to sustainable health and well-being over the lifespan. The analysis of health-related behaviors is crucial for understanding the state of health in different populations, especially teachers who play a critical role in establishing the lifelong health behaviors of their pupils. This multidisciplinary, nationwide study aimed to assess and compare lifestyle patterns of Austrian teachers and school principals at secondary levels I and II with a specific focus on physical activity and diet. A total number of 1350 teachers (1.5% of the eligible Austrian sample; 69.7% females; 37.7% from urban areas; mean age: 45.8 ± 11.4 years; mean BMI: 24.2 ± 4.0) completed a standardized online survey following an epidemiological approach. Across the total sample, 34.4% were overweight/obese with a greater prevalence of overweight/obesity in males than females (49.5% vs. 29.2%, *p* < 0.01) and rural vs. urban environments (35.9% vs. 31.3%). Most participants (89.3%) reported a mixed diet, while 7.9% and 2.9% were vegetarians and vegans, respectively. The average BMI of teachers with mixed diets (24.4 ± 4.0 kg/m^2^) was significantly higher than vegetarians (23.1 ± 3.2 kg/m^2^) and vegans (22.7 ± 4.3 kg/m^2^). Vegans reported a lower level of alcohol intake (*p* < 0.05) among dietary groups. There was no between-group difference in smoking (*p* > 0.05). The prevalence of engagement in regular physical activity was 88.7% for leisure-time sports/exercises and 29.2% for club sports. Compared with the previous reports on general populations, the present data suggest an acceptable overall health status among Austrian teachers.

## 1. Introduction

Despite the generally good health status in the Austrian adult population, some indicators show an increased risk for health and potential threats in future years [1]. A report from the Organization for Economic Cooperation and Development (OECD) also highlights that the main risk factors for such situations are behavioral parameters, such as poor diet, unhealthy habits, and low physical activity (PA) [1]. In this regard, the OECD adds that, while life expectancy is similar to the rest of the EU, a more detailed analysis shows that Austrians spend fewer years free of disabilities (57 years compared with the average EU of 64 years) and more years with chronic diseases or disabilities compared with their European peers. Indeed, the role of daily PA and a healthy diet as promoters of health in preventing diseases has been underlined in several studies over the past several decades [2,3].

The beneficial effects of PA on several health threats have been well-documented [2]. It is well-accepted that healthy activity patterns and increased levels of PA, sports, and exercise result in higher cardiorespiratory fitness, and consequently, an improved level of health [4]. Regular exercise may be responsible for several benefits in all dimensions of health, including an increase in cognition even in the presence of neurodegeneration [5], prevention of stress and depression [6,7], maintenance of a good health profile even in adverse situations [8], and lower risk of developing discomforts in individuals who sit for prolonged amounts of time [9]. PA can be carried out in different ways and is categorized mostly as leisure recreational exercise (independent of commercial suppliers such as sports units, clubs, federations) or by participating in sports activities at a professional or amateur level (usually through membership in sports clubs). According to the literature, both types hold a strong relationship with health: the former is associated with better self-rated health and reduced sick days in workers [10], lower cardiovascular mortality and incidence of metabolic syndrome [11], and even improved quality of sleeping [12]; the latter triggers improved health profile and well-being [13], promotes physical fitness [14], and increases aerobic capacity and muscular strength resulting in favorable cardiovascular health outcomes [15].

According to current data from the Global Burden of Disease Study [16], 20% of Western Europeans die prematurely due to poor dietary habits and unhealthy diets mainly characterized by the overconsumption of meat, processed meat, and salt, as well as from insufficient intake of fruits, vegetables, whole grains, legumes, nuts, and seeds. Adherence to healthy diets seems to be associated with a better weight-related profile (i.e., lower body mass index (BMI) and waist circumference) [17]. While certain diet types are associated with a lower incidence of cancer [18], diet quality is an important predictor of non-communicable diseases and, therefore, could be considered an effective preventive intervention [19]. In general, healthier diets may lead to higher overall health [20]. Although vegetarian and vegan diets are well-documented to promote and maintain good health and to prevent and treat various severe health conditions [21,22], Bloomer et al. [23] suggest that both vegan and omnivorous diets, when designed appropriately, may improve several metabolic parameters associated with health, such as blood pressure and lipidic profile. In general, however, evidence shows that each type of diet implies benefits and risks, and dietary advice is recommended in order to fill potential nutritional gaps [24].

From the above-mentioned, the analysis of PA and diet type, especially their continuous interaction, along with sociodemographic and individual factors intervening in such interactions (e.g., BMI [25], living area [26], or sex [27]), becomes essential for understanding the health status of any population. The necessity of these topics is more remarkable for specific populations, such as teachers, who are considered crucial role models for children and adolescents not only for teaching healthy behaviors but generally due to their strong impact on youth lifestyle choices [28,29]. In fact, teachers have been shown to be an essential element in increasing pupil PA behavior [30] and play a predominant role in enhancing children’s health alongside parents [31]. Indeed, it is suggested that pupils tend to show higher PA rates if their teachers engage in exercise [28]. Furthermore, they are considered important in triggering pupil behavioral change towards better well-being [32]. A study investigating the effect of a program of teacher professionalism and literacy in nutrition on pupil nutritional habits and choices confirms a strong association between them, as pupils whose teachers participated in the intervention significantly increased their knowledge about healthy diets [33].

Health promotion through healthy lifestyles, including exercise/sports and healthy nutrition, is a state mandate of the Austrian curriculum at primary and secondary school levels and is defined as an overarching educational goal [34,35,36,37,38]. To date, only a limited number of reports focusing on the health of teachers [39,40,41,42] and school managers [43,44] exist, mostly with a major focus on mental health and stress management considering the school environment. There is a gap in the literature on the association between exercise behaviors and diet types (especially vegetarian, vegan) in the school context, except for one current report from our laboratory on secondary pupils [45]. Therefore, the present study aimed to examine the lifestyle behaviors of a large group of Austrian teachers and school principals at secondary levels I and II with a first glance not only to provide descriptive epidemiological data but a specific focus on “PA/sport participation” and “diet type” subgroups to identify potential associations.

## 2. Materials and Methods

### 2.1. Study Protocol and Ethical Approval

*From Science 2 School* (www.science2.school/en, accessed on 8 February 2022) was designed as a cross-sectional study with a multidisciplinary approach using a multi-level cluster sampling strategy. This study was conducted nationwide in Austria with a large sample and was supported by the Federal Ministry of Education, Science, and Research, Department 1/7—School and University Sports. The study protocol was approved by the ethical board of all nine Federal Education Authorities in Austria (*Bildungsdirektionen*), which was mandatory in order to contact the principals of 2688 secondary schools (levels I and II) across Austria to commence the study. Due to the requirements of the respective Austrian federal education authorities and the respective school management, no further ethical vote (e.g., local ethical committee or the institutional review board) was required for the present study. Interested readers are kindly referred to the study protocol [46].

### 2.2. Participants

**Figure 1:**
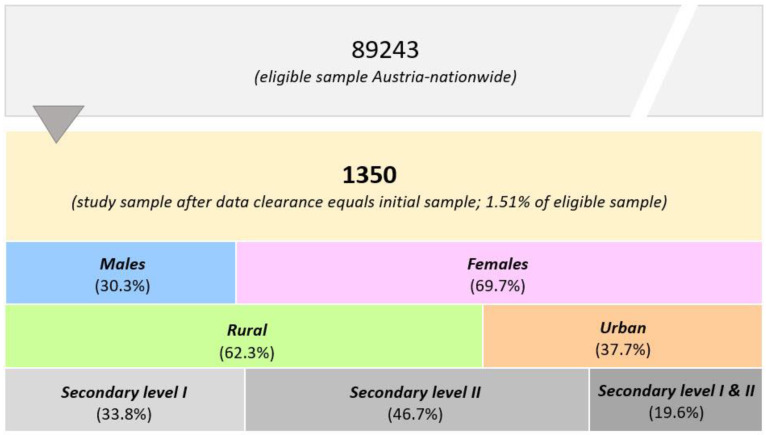
Flow chart of the participants’ enrollment and classifications by sex (males vs. females), living area (rural vs. urban), and school level.

The target group was all teachers and principals of secondary school levels I and II, resulting in a total sample size of 89,243 adults. Accordingly, all secondary schools (levels I and II: *n* = 2688) were invited to participate in the study. At the closure of data collection (10 July 2020), a total of 1350 teachers and principals (1.5% of eligible participants) participated in the online survey. Participants’ enrollment is shown in Figure 1.

### 2.3. Procedures

The school management received (i) comprehensive initial information about the study via email and subsequently by personal communication, such as a telephone call (goal and procedure of the study), and (ii) material (cover letter for teachers/class directors, the respective web links for the online survey for adults vs. pupils, and information letter for parents/guardians). The principal transferred this information and materials to all teachers within their respective teaching staff to participate in the survey.

Secondary-level teachers and school principals who intended to contribute to the study completed a standardized online questionnaire via an encrypted interface (available/provided in German). Prior to filling out the questionnaire, participants received written information about the study procedure (which was provided online) and gave their written informed consent to participate in the study. Participation was voluntary and anonymous and could be withdrawn by the participant at any time without the provision of reasons. Participants completed the online questionnaire via smartphone, tablet, or PC/laptop.

The survey consisted of five parts with questions about sociodemographic characteristics (part 1), PA and sports (part 2), nutrition (part 3), health (part 4), and miscellaneous (part 5). Control questions were included in different parts of the questionnaire to identify conflicting data and increase the reliability of data sets.

Regarding the operational implementation, three steps were required: (1) approval of the survey in all nine Austrian federal states by the respective state school boards of each federal educational authority; (2) approval of the questionnaire to be implemented directly in the schools by the acting school management in all nine Austrian federal states; and (3) support by the Federal Ministry of Education, Science, and Research to facilitate contact and the procedure of the study with the participating schools. 

**Figure 2:**
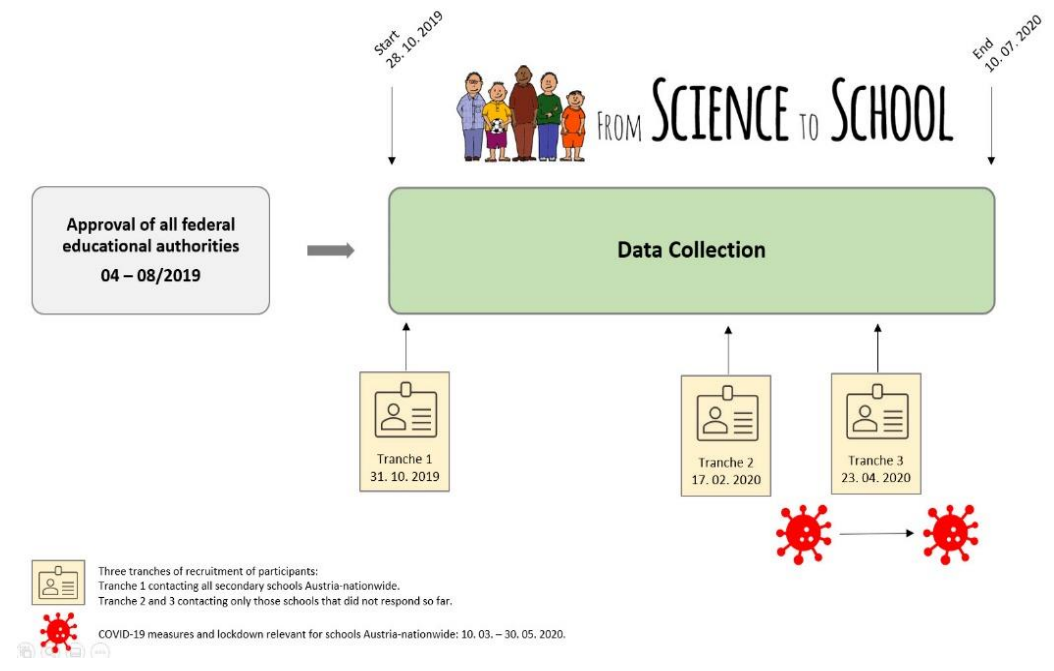
Flow chart of procedure and timescale of the application for approval of educational authorities and subsequent data collection by online survey.

Figure 2 displays the procedure (recruitment of participants accomplished by three tranches) and timescale of the previous approval by educational authorities and the subsequent data collection via the online survey.

### 2.4. Measures

The survey collected self-reported data on socio-demography (age, sex, nationality, federal state, living area, and region); anthropometry (body weight, height, calculated BMI (kg/m^2^)); level and type of secondary school; nutrition (e.g., fruit and vegetable, fluid, current diet type), including alcohol consumption; smoking prevalence; and PA. Based on the diet type reported, participants were categorized into vegetarian (devoid of meat and processed meat inclusive fish and shellfish, but intake of dairy and eggs), vegan (devoid of all foods and ingredients from animal sources), or omnivore/mixed dietary subgroups (no dietary restrictions). For PA, participants reported their engagement in leisure time activities in sports and exercise (e.g., type of activity, duration/day, frequency/week, organizational form, competition participation, member of a sports club).

Participants with a body weight of <20 kg, height < 110 cm, or those with calculated BMI values of <10 kg/m^2^ or >50 kg/m^2^ (deemed implausible) were removed from data analysis.

### 2.5. Data Clearance

All adults who participated (1350) were included in the final data analysis (1.5% of the eligible 89,243 teachers/principals) since all anthropometric data available were plausible after a review of the raw data (data clearance). Further detailed information is available in the study protocol [46].

### 2.6. Statistical Analysis 

Descriptive statistics were calculated, and data were reported as mean (±standard deviation) for continuous data and prevalence for nominal data. Differences in anthropometric characteristics by living environment (urban vs. rural), sex, school level, sports participation, and nutrition were examined via multivariate analysis of variance (MANOVA). Differences in sports participation and dietary pattern by living environment were examined via chi-square tests. Further, chi-square tests were used to examine differences in dietary pattern (e.g., intake of fruits, vegetables, and fluids), smoking and alcohol use by sports participation, and diet type (mixed vs. vegetarian vs. vegan diet). Chi-square tests were used to examine differences in sports participation by diet type. All statistical tests were conducted by SPSS 26.0 (SPSS Inc., IBM Corp., Armonk, NY, USA). The statistical significance level was set at *p* ≤ 0.05.

## 3. Results 

A total of 1350 (30.3% male, 69.7% female) secondary level I and/or II teachers from all nine Austrian federal states completed the questionnaire. Participants were between 20 and 65 years of age and have taught between 1 and 46 years (mean 19.4 ± 12.5 years). 

The distribution of participants by their living area, nationality, and workplace is shown in Table 1. Almost half of the participants (46.7%) taught at secondary school level II, and one-third (33.8%) taught at secondary school level I. The remainder of the participants taught at both secondary school levels. Across the entire sample, 62.3% lived in rural areas, and 97.6% were Austrian. Among non-Austrian participants, the majority came from Germany (1.3%), followed by Italy and Hungary (0.2%) as well as Denmark, France, Greece, Croatia, Romania, Bosnia and Herzegovina, Serbia, Egypt, and the USA (0.1%).

### 3.1. Anthropometric Characteristics

Anthropometric characteristics in the total sample and based on sex and school type (secondary school level I, secondary school level II, both) are shown in Table 2. Supplementary results provided in Appendix A Table A1 show anthropometric data by federal state separately for urban and rural areas. Male teachers were significantly older than female teachers (*p* < 0.01). As expected, men were taller and heavier than women, but they also had a higher BMI (*p* < 0.01). Accordingly, there was a sex difference in the prevalence of overweight and obesity, indicated by a BMI above 25 (*p* < 0.01). Almost half of the men (46.5%) but less than one-third of the women (29.2%) were considered overweight/obese. The prevalence of underweight was significantly higher in women compared with men (*p* < 0.01). Across the total sample, there were no significant differences in age or anthropometric characteristics between teachers in urban or rural environments. Similarly, anthropometric characteristics did not differ between teachers teaching in secondary school level I, secondary school level II, or both.

### 3.2. Sports Participation

The distribution of sports participation is presented in Table 3, while Table 4 shows anthropometric characteristics by sports participation. A majority of participants (88.7%) reported taking part in regular PA or sports during their leisure time, but less than one-third of the participants (29.2%) were active in sports clubs. There was no sex difference in leisure-time sports participation. More men engaged in club sports compared with women (39.1% vs. 24.9%; *p* < 0.01). Across the entire sample, participants engaged in sports over 2.9 ± 1.5 days a week with a higher sports participation in males than females (*p* = 0.02). The prevalence of club sports participants was also higher in rural compared with urban participants (*p* = 0.02), but there was no difference in the number of days per week participants engaged in sports by living environment. Leisure-time sports participation was significantly associated with lower body weight and BMI (*p* < 0.01). Accordingly, the prevalence of overweight and obesity was significantly lower in leisure-time sports participants compared with non-sports participants (32.0% vs. 53.6%; *p* < 0.01) even though the prevalence of underweight was higher in non-sports participants (*p* = 0.02). Despite no significant difference in BMI between club sports participants and those not engaging in club sports, non-club sports participants had a higher obesity prevalence (*p* = 0.03).

### 3.3. Diet

Diet type distributions are displayed in Table 5, while Table 6 shows anthropometric characteristics by kind of diet. Based on the self-reports, most participants (89.3%) consumed a mixed diet. A mixed diet was more common in men compared with women, while a vegetarian diet was more common in women compared with men. Only a small number of the participants reported a vegan diet, and there was no difference between men and women. Despite being the dominant diet type in both urban and rural areas, a mixed diet was even more common in rural areas due to a lower prevalence of vegan diets. There was no difference in vegetarian diet patterns between urban and rural living situation. No differences in dietary pattern were observed across teaching levels or by nationality. Participants reporting a mixed diet had a significantly higher BMI than their peers with a vegetarian or vegan diet. Nevertheless, the prevalence of overweight and obesity did not differ across dietary patterns. The prevalence of underweight was significantly higher in participants adhering to a vegan diet (*p* < 0.01).

### 3.4. Physical Activity and Health Behaviors

Across the entire sample, 62.4% reported daily fruit intake, and 72.2% reported daily vegetable intake. The majority of participants (76.1%) reported water as the most commonly consumed fluid, but only 27.0% drank more than 2 L/day. A total of 81.5% consumed alcohol, and 11.0% were smokers. Table 7 shows the association between sports participation and health behaviors. Compared with non-sports participation, leisure-time sports participation was associated with a higher prevalence of daily fruit and vegetable consumption (*p* < 0.01) as well as a higher likelihood of fluid intake above 2 L/day (*p* < 0.01). The prevalence of daily fruit and vegetable consumption was also higher in participants reporting leisure-time sports (*p* < 0.01). Furthermore, the prevalence of daily fruit and vegetable intake and fluid consumption above 2 L/day increased with a higher weekly frequency of engagement with sports (*p* < 0.01). Even though there was no significant difference in alcohol consumption by days of sports participation, leisure-time sports participation was associated with a higher prevalence of alcohol consumption (*p* = 0.03). Smoking rates were lower in participants reporting leisure-time sports and declined further with the increasing number of days engaging in sports (*p* < 0.01). The association of club sports participation with dietary habits was limited to a significantly higher total fluid intake in club sports participants compared with non-club sports participants (*p* < 0.01).

Diet types did not differ by participation in leisure-time or club sports (Table 8). There was also no difference in the prevalence of fluid intake above 2 L/day, alcohol consumption, or smoking between participants with a mixed, vegetarian, or vegan diet. Water as the most common fluid was more prevalent with a vegetarian diet, while participants with a vegan diet were least likely to report water as the most common fluid (*p* < 0.01).

Supplementary information on sports participation, eating behaviors, alcohol consumption, and smoking prevalence by federal state and living area is provided in Appendix A Table A2.

## 4. Discussion

The most important health-promoting characteristics, considered as powerful indicators of health, include nutrition and PA, which not only apply to teachers but also to every population [44,46]. This study aimed to examine the lifestyle behaviors of a large group of Austrian teachers and principals at secondary schools (levels I and II) with a specific focus on “PA/sport” and “diet” across different subgroups.

An overview of the results from the present study indicates that teachers seem to have a healthier lifestyle (associated with BMI, PA pattern, alcohol intake, smoking) compared with general populations reported by similar investigations [40]. This general finding could be linked to an Austrian health study reporting teachers to categorize themselves as good-to-excellent in the overall state of health [39]. Evidence shows that the health of a school’s teaching staff has a significant impact on the quality of teaching and thus also on the pupils’ learning success [47] with the (teaching) quality of teachers being the most important factor in the success of education systems [48]. Health, in general, is closely related to action competence and personality development [44] with positive attitudes to healthy lifestyles and behaviors shown to track over time from childhood to adulthood and old age [46,49]. Teacher health is not only a prerequisite for high-quality education but is crucial for successful societies, while healthy teachers positively contribute to educating and growing healthy children via a distinct impact on pupils’ lifestyle choices [28,29]. The promotion of teacher health is thus not a “private matter” of individual teachers but a contribution to the education system and the general public as a whole [44] in terms of public health issues for nations such as Austria.

Contrary to previous studies, the present findings showed that male teachers displayed higher obesity and overweight levels than their female counterparts. This result is in line with an Austrian survey on teachers within the framework of the Health Behavior of School-Aged Children (HBSC) report 2010 [40,41], where 60.6% of teachers were found to have a normal BMI (vs. 63% in the present study), and overweight and obesity among teachers was considerably higher in males than females (40.6% vs. 14.7%). While the fraction of teachers in BMI_NORM_ is markedly higher than the Austrian normal-weight general population (49.8%), this positive trend is evident with overweight (29.1% vs. 35.3%) and obese (8.2% vs. 12.4%) teachers compared with the general population, too. It can be suggested that while nutritional changes may have affected both sexes, negative changes in PA habits were more common in males, while females are well-accepted to be more health conscious, which might have led to such differences [50,51,52]. However, Kanter and Caballero [53] reported increased obesity rates in both males and females of the general population, and the increase was significantly higher in women. According to Ameye and Swinnen [54], women present higher obesity rates in low- and middle-income countries, whereas sex differences become null in high-income countries. Furthermore, the Centers for Disease Control and Prevention [55] report that in the adult population, sex differences are only significant for severe obesity, which is more common in women (11.5%) than men (6.9%), and overall obesity incidence is equal for males and females (40.3% and 39.7%, respectively). However, our findings on the underweight category are in accordance with the literature available included in the HBSC report [40,41] on teachers’ health (underweight prevalent in females only), as women tend to present such conditions more commonly than men. Although the prevalence of underweight seems to have decreased over the last two decades, the percentage of underweight individuals is higher in females (9.7%) than males (8.8%), according to a longitudinal observation study carried out internationally since 1975 [56]. Similar to our findings, a higher prevalence of underweight in females is also reported by Zhang et al. [57] and other recent research on adult populations from different regions of the world [58,59]. While different reasons have been reported to trigger underweight conditions, dietary-related causes are known to pose higher health risks [60].

PA level in Austrian adults is known to be generally higher than recommended, as stated by the OECD report [1] and other scientific sources [61,62]. This discovery could be consistent with the present findings in terms of leisure-time physical activities but not sports club sport/exercise. In the present study, the average amount of sport days/week is lower than the recommended amount by international health resources [63,64,65] indicating weekly PA, sports, and exercise should be at least 150 min of moderate-to-vigorous activity or 75 min of vigorous activity, preferably daily (approx. 20 min) [66] over 4 to 5 days per week. However, the teachers in the present study are physically active over slightly less than three days per week on average. With this lower than recommended weekly frequency of PA, it seems less likely (or at least more difficult) to achieve the recommended duration of weekly PA necessary for health benefits. However, this cannot be fully confirmed with the data from the present study, as the PA questionnaire implemented did not collect information on the duration or intensity of teachers’ exercise bouts. HBSC reports show that both female and male Austrian teachers were more active than general populations [40,41]; however, they were less active than female and male teachers in the present findings in terms of leisure-time PA. 

Of particular interest is that the differences in weight status and BMI could be partially linked to participation in leisure-time PA rather than participation in sports club activities. Although sports club participation was associated with a significantly higher percentage of normal-weight individuals and a significantly lower rate of obesity, this difference was null for two other health-related categories (i.e., underweight and overweight). The type of engagement in PA, however, appears to be even more critical, as engagement in leisure-time sports was more strongly associated with weight profile (from the lower total weight and BMI scores to lower rates of underweight, overweight, and obesity, and higher rates of normal weight). According to Su et al. [67], leisure-time PA is an independent factor positively affecting weight control and obesity/overweight rates and risk; the benefit of leisure-time PA is not affected but also does not affect the consequence of sedentary time. Quist et al. [68] suggest that active commuting to different places (workplace, school, etc.) is an essential component of leisure-time PA that may lead to better weight profile over the time. This report is confirmed by Schäfer et al. [69], who carried out a literature review on active commuting in Austrian populations and reported that actively commuting had a positive impact on cholesterol, lipid profile, waist circumference, and other weight-related variables. Considering that active commuting is spread in Austria, we might argue that this type of leisure-time PA may have contributed to our findings [70]. In addition, dietary intake was associated with leisure-time PA in the present sample with those stating engagement in leisure-time PA reporting a significantly higher intake of fruit and vegetables, which increased even more for those exercising at least 4 days per week, i.e., matching the recommendations for healthy PA. In the present study, however, no remarkable difference was observed between dietary groups (omnivores, vegetarians, vegans) in terms of PA engagement. The close connection between exercise and healthy food choices has been demonstrated by previous studies [71,72,73], which seems to be associated with better knowledge and awareness of health and with perceiving a healthy diet and healthy PA guidelines as useful and realistic [74].

Our participants’ nutritional profile showed a major preference for a mixed diet, as almost 90% of the sample reported being omnivores. The relationship between diet type and weight profile in our sample further pointed out that an omnivorous choice is associated with a lower percentage of underweight but a higher percentage of overweight than vegan teachers, along with a higher rate of obesity compared with vegetarians. On the contrary, vegetarians had a better ratio of normal weight and obesity compared with the other diet types, whereas those adhering to vegan diet had a significantly better BMI, but at expense of normal weight, and were found to have a higher percentage of underweight. Similar health patterns across diet types were also reported in a study on Austrian pupils where vegetarians showed lower obesity rates yet a higher percentage of underweight adolescents [46]. The present finding is also consistent with previous research that showed lower body weight and BMI in vegans compared with vegetarians and omnivores [75,76], and consequently, a lower prevalence of overweight and obesity [77]. This outcome might be justified by the fact that people who follow vegetarian or vegan diets are known to be more health-conscious (more active and less consumption of alcohol) [78]. A randomized controlled trial on adults aged 18 to 65 years investigating the effect of different diet types on weight reported that plant-based diets were generally more efficient than the mixed diet for weight loss [75]. These findings are supported by Medawar et al. [76], who state that vegetarian and vegan diets should be deemed as important strategies in the attempt to reduce obesity and overweight as effective weight-loss approaches. In addition, a longitudinal study on nearly 90,000 adults whose dietary habits and anthropometric data were measured for 6.5 years demonstrated a statistically significant link between consumption of animal proteins and long-term weight gain [79], further supporting our findings on lower underweight and higher obesity rates in those who adhere to a mixed diet compared with vegans and vegetarians. The association between the mixed diet and weight gain has also been suggested by Tucker et al. [80]. However, it should be generally considered that irrespective of diet type, diet quality and food components (which were absent from investigation in the present study) have been shown to be remarkable predictors of health and weight management [19], and thus, caution must be warranted when interpreting the findings. Regardless of this fact, plant-based diets have a significant weight-loss effect, which in general terms could be considered positive in those individuals who need to reduce their weight for health purposes [78,79]. Nonetheless, this decrease may also present risks in an unintended underweight condition. Being in a severe underweight condition is associated with a series of health conditions, for instance, higher odds of developing renal pathologies as a comorbidity of diabetes type 2 [81], osteoporosis, anemia and inefficient immune system [82], or fertility issues [83].

The latest health reports on Austrian teachers [39,40,41] pools health-related lifestyle behaviors such as PA, diet, smoking, etc. within the item “lifestyle” rather than distinctly discriminating activity patterns and dietary types or trends from other lifestyle factors such as smoking and alcohol consumption. Findings from the HBSC teachers report 2010 [40,41] neither link activity patterns to various diets nor display current nutritional trends and diet types, and also report no information on alcohol consumption. Regarding smoking, the HBSC study showed that teachers had a lower prevalence of daily smoking than the general public (12.6% vs. 23.3%), with most teachers quitting with age [40,41]. While vegans are generally known to be more health-conscious (smoking and drinking alcohol less, more active) [71,78], vegetarian teachers in the present study had the lowest prevalence of smoking and alcohol intake among dietary groups.

The present study may be considered a pioneer in studying current levels of PA, sports, and exercise linked to the prevalence of various diet types among Austrian adults, but especially teachers as a professional group that constitutes a role model for the young populations. However, some limitations should be mentioned: (1) the design is cross-sectional, limiting the possibility to assess any cause–effect relationship; (2) the likelihood of socially desired over-reporting (e.g., higher consumption of healthy food items, longer duration of PA) or under-reporting (e.g., lower body weight, taller height, lower consumption of unhealthy food items); (3) although the application of BMI provides a high predictive validity and is proposed as a more flexible view of the measure as a holistic appraisal of health [84], and thus, an accurate indication of fat mass in adults, this assessment method is known to present some flaws, including inability to directly measure body fat and lean mass [65,85]; (4) the assessment method for PA does not clarify essential aspects of exercise habits, such as intensity or total weekly duration, which are both important to establish the real impact of PA on health; (5) although our study is the first delving in the association between diet types and PA, there exists a variety of nuances in each of the types assessed (omnivorous, vegetarian, vegan), as well as completely different dietary approaches not included in this study; and (6) although the Austria-wide sample allows for the generalization of results for Austria, which could be comparable with countries with similar culture and geography such as Germany and Switzerland (i.e., the D-A-CH countries), factors such as socio-environmental characteristics, culture, and different school systems may affect lifestyle-related outcomes. In addition, the current pandemic of COVID-19 (inclusive lockdowns) might have affected the public schools and universities in the later leg of the study with measures put into action in March 2020; it was not possible to take into account this unpredictable COVID-19 situation within the online survey without any consequence that could have potentially affected the data collection and accuracy (e.g., loss of data due to stopping and restarting the online survey, biased data, conflicting data sets of prior vs. during vs. post COVID-19 situation, etc.). 

## 5. Conclusions

The present study provides epidemiological information regarding lifestyle behaviors of 1350 Austrian secondary-level schoolteachers and principals. In general, Austrian teachers/principals had a healthier lifestyle (in terms of BMI, diet type, PA level, smoking, and alcohol intake) compared with general populations reported by similar investigations. This finding might be due to the higher educational level of teachers, particularly the fact that they have more individual capabilities (including knowledge, skills, competencies, qualifications, values) as well as social advantages (e.g., networks, general living conditions) to implement toward scientifically well-accepted healthy behaviors. However, more attention should be considered regarding the disadvantageous lifestyle behaviors of teachers found in the present study (e.g., the lower level of teachers’ PA than international recommendations, moderate level of fruit and vegetable intake) as they are considered as role models and front-line educators and multipliers of the next generations.

The present data emphasize the need for continued efforts to facilitate healthy lifestyle choices among Austrian teachers and principals. PA adherence seems not to be related to a particular nutritional choice, which reinforces the idea that all types of diet may work well in synergy with active behaviors; however, adherence to exercise across the three diet types is strongly associated with healthier nutritional choices in terms of vegetables and fruit intake. Increasing PA levels along with adhering to healthful diet types (e.g., whole food plant-based diets), which is known as the dual approach of “healthy eating–active living,” is recommended from our lab as a minimum recommendation to achieve lifelong and sustainable health, and consequently, to improve public health.

## Figures and Tables

**Table 1:** Sample distribution. Values are number of participants (N) and prevalence (%).

	Total (N)	Male N (%)	Female N (%)	Secondary Level I N (%)	Secondary Level II N (%)	Secondary Level I & II N (%)
	1350	409 (30.3)	941 (69.7)	456 (33.8)	630 (46.7)	264 (19.6)
**Living Environment**						
Urban	509	150 (29.5)	359 (70.5)	126 (24.8)	247 (48.5)	136 (26.7)
Rural	841	259 (30.8)	582 (69.2)	330 (39.2)	383 (45.5)	128 (15.2)
**Nationality**						
Austrian	1318	398 (30.2)	920 (69.8)	446 (33.8)	619 (47.0)	253 (19.2)
Other	32	11 (34.4)	21 (65.5)	10 (31.3)	11 (34.4)	11 (34.4)
**School Type**						
Middle School	485	133 (27.4)	352 (72.6)	X		
AHS	216	80 (37.0)	136 (63.0)	X	X	X
Middle School and AHS	6	3 (50.0)	3 (50.0)	X		X
Prevocational School	44	23 (52.3)	21 (47.7)		X	
AHS and BMS	117	37 (31.6)	80 (68.4)		X	
AHS and BHS	22	4 (18.2)	18 (81.8)		X	X
Sec. Tech. and Voc. School (BMS, 4 years)	86	33 (38.4)	53 (61.6)		X	
Sec. Tech. and Voc. School (BHS, 5 years)	313	91 (29.1)	222 (70.9)		X	
Other	61	5 (8.2)	56 (91.8)	X	X	X

Sec. I—secondary school level I; Sec. II—secondary school level I; Sec. Tech. and Voc. School—secondary technical and vocational school; AHS—academic school (Allgemeinbildende Höhere Schule: Sec. I, Sec. II, or Sec. I and Sec. II combined since separation is not possible at AHS); BMS—Berufsbildende Mittlere Schule (4 years); BHS—Berufsbildende Höhrere Schule (5 years). X—Type of school grouped in either secondary school level I, school level II, or both Sec. I and Sec. II combined).

**Table 2:** Anthropometric characteristics for the total sample and separately for male and female participants, secondary level I, secondary level II, and both secondary level I and II combined. Values are means ± SD and prevalence for weight categories.

	Total	Male	Female	Secondary Level I	Secondary Level II	Secondary Level I and II
**Age (years)**	**45.8 ± 11.4**	**47.3 ± 11.4**	**45.2 ± 11.4**	**45.6 ± 12.0**	**46.7 ± 11.0**	**43.9 ± 11.3**
Urban	45.9 ± 11.6	46.2 ± 11.7	45.8 ± 11.6	46.2 ± 12.5	47.0 ± 11.3	43.7 ± 11.2
Rural	45.7 ± 11.3	48.0 ± 11.1	44.7 ± 11.2	45.4 ± 11.8	46.6 ± 10.7	44.2 ± 11.5
**Body Weight (kg) ^1,2^**	**71.3 ± 14.6**	**82.6 ± 12.6**	**66.4 ± 12.5**	**70.4 ± 14.3**	**71.9 ± 15.0**	**71.4 ± 14.0**
Urban	70.5 ± 14.0	80.8 ± 12.5	66.1 ± 12.3	70.5 ± 13.7	70.4 ± 14.4	70.6 ± 13.8
Rural	71.8 ± 14.9	83.6 ± 12.5	66.5 ± 12.6	70.4 ± 14.5	72.8 ± 15.3	72.3 ± 14.3
**Height (cm) ^2^**	**171.2 ± 8.3**	**179.8 ± 6.7**	**167.4 ± 5.8**	**170.4 ± 7.8**	**171.6 ± 8.7**	**171.6 ± 8.2**
Urban	171.0 ± 8.1	179.5 ± 6.9	167.5 ± 5.5	170.8 ± 8.0	170.7 ± 8.2	171.9 ± 7.9
Rural	171.3 ± 8.5	180.0 ± 6.6	167.4 ± 5.9	170.2 ± 7.7	172.2 ± 9.0	171.3 ± 8.4
**BMI (kg/m^2^) ^1^**	**24.2 ± 4.0**	**25.5 ± 3.4**	**23.6 ± 4.1**	**24.2 ± 4.1**	**24.3 ± 3.9**	**24.2 ± 3.9**
Urban	24.0 ± 3.9	25.0 ± 3.3	23.5 ± 4.1	24.1 ± 3.9	24.0 ± 3.9	23.8 ± 3.9
Rural	24.3 ± 4.0	25.8 ± 3.4	23.7 ± 4.1	24.2 ± 4.2	24.4 ± 3.9	24.5 ± 3.9
**Underweight (%)**	**2.6**	**0.0**	**3.7**	**3.1**	**2.5**	**1.9**
Urban	3.0	0.0	4.1	2.4	3.2	3.0
Rural	2.4	0.0	3.4	3.3	2.1	0.8
**Normal Weight (%)**	**63.0**	**53.5**	**67.1**	**61.6**	**62.1**	**67.7**
Urban	65.2	59.3	67.6	61.1	64.8	69.6
Rural	61.7	50.2	66.8	61.8	60.3	65.6
**Overweight (%)**	**25.6**	**36.2**	**21.0**	**26.5**	**27.3**	**19.8**
Urban	22.6	31.3	19.8	29.4	23.5	17.0
Rural	27.0	39.0	21.6	25.5	29.8	22.6
**Obese (%)**	**8.8**	**10.3**	**8.2**	**8.8**	**8.1**	**10.6**
Urban	8.7	9.3	8.4	7.1	8.5	10.4
Rural	8.9	10.8	8.1	9.4	7.8	10.9

BMI—body mass index. Bold—total numbers. ^1^ Significant difference between male participants living in urban and rural areas (*p* < 0.05); ^2^ significant difference between secondary-level II participants living in urban and rural areas in (*p* < 0.05).

**Table 3:** Sports participation by sex, teaching level, living area, and nationality. Values are number of participants (N) and prevalence (%) and mean with standard deviation for number of days with sports.

	Leisure-Time Sports N (%)	Club Sports N (%)	Sport Days/Week Mean ± SD
**Total Sample**	**1198 (88.7)**	**384 (29.2)**	**2.9 ± 1.5**
Male	372 (91.0)	160 (39.1)	3.1 ± 1.5
Female	826 (87.8)	234 (24.9)	2.9 ± 1.4
**Teaching Levels**			
Secondary Level I	408 (89.5)	139 (30.5)	2.9 ± 1.4
Secondary Level II	556 (88.3)	167 (26.5)	2.9 ± 1.5
Both (Level I and II)	234 (88.6)	88 (33.3)	3.1 ± 1.5
**Living Environment**			
Urban	452 (88.8)	130 (25.5)	2.9 ± 1.4
Rural	746 (88.7)	264 (31.4)	3.0 ± 1.5
**Nationality**			
Austria	1170 (88.8)	387 (29.4)	2.9 ± 1.4
Other	28 (87.5)	7 (21.9)	3.1 ± 1.6

**Table 4:** Anthropometric characteristics by sports participation. Values are means ± SD and prevalence for weight categories.

	Leisure-Time Sports		Club Sports	
	Yes	No	Yes	No
**Age (years)**	45.8 ± 11.5	45.8 ± 10.9	45.7 ± 11.4	45.9 ± 11.4
**Height (cm) ^2^**	171.2 ± 8.2	171.1 ± 9.1	172.7 ± 8.3	170.6 ± 8.2
**Body Weight (kg) ^1^**	70.7 ± 13.9	76.1 ± 18.2	71.8 ± 14.0	71.1 ± 14.8
**BMI (kg/m^2^) ^1^**	24.0 ± 3.7	25.9 ± 5.4	24.0 ± 3.6	24.3 ± 4.1
**BMI Subgroups (%)**				
Underweight (%) ^1^	2.3	4.6	1.5	3.0
Normal weight (%) ^1,2^	65.7	41.7	68.8	60.6
Overweight (%) ^1^	24.5	34.4	23.1	26.6
Obese (%) ^1,2^	7.5	19.2	6.6	9.7

^1^ Significant difference by leisure-time sports participation (*p* < 0.05); ^2^ significant difference by club sports participation (*p* < 0.05).

**Table 5:** Diet type by sex, teaching level, living area, and nationality. Values are number of participants (N) and prevalence (%).

	Mixed Diet N (%)	Vegetarian N (%)	Vegan N (%)
**Total Sample**	**1205 (89.3)**	**106 (7.9)**	**39 (2.9)**
Male	384 (93.9)	14 (3.4)	11 (2.7)
Female	821 (87.2)	92 (9.8)	28 (3.0)
**Teaching Levels**			
Secondary Level I	399 (87.5)	44 (9.6)	13 (2.9)
Secondary Level II	568 (90.2)	45 (7.1)	17 (2.7)
Both (Level I and II)	238 (90.2)	17 (6.4)	9 (3.4)
**Living Environment**			
Urban	442 (86.8)	46 (9.0)	21 (4.1)
Rural	763 (90.7)	60 (7.1)	18 (2.1)
**Nationality**			
Austria	1177 (89.3)	103 (7.8)	38 (2.9)
Other	28 (87.5)	3 (9.4)	1 (3.1)

**Table 6:** Anthropometric characteristics by dietary pattern. Values are means ± SD and prevalence for weight categories.

	Mixed Diet	Vegetarian	Vegan
**Age (years) ^2^**	45.9 ± 11.4	45.0 ± 10.7	44.2 ± 12.3
**Height (cm) ^1^**	171.3 ± 8.3	169.2 ± 6.9	172.2 ± 10.9
**Body Weight (kg) ^1^**	71.8 ± 14.5	66.4 ± 11.1	71.3 ± 14.6
**BMI (kg/m^2^) ^1,2^**	24.4 ± 4.0	23.1 ± 3.2	22.7 ± 4.3
**BMI Subgroups (%)**			
Underweight (%) ^1,2^	2.2	2.8	15.4
Normal Weight (%) ^2,3^	62.6	69.8	56.4
Overweight (%)	25.9	23.6	20.5
Obese (%) ^1,3^	9.3	3.8	7.7

^1^ Significant difference between mixed diet and vegetarian diet (*p* < 0.05); ^2^ significant difference between mixed diet and vegan diet (*p* < 0.05); ^3^ significant difference between vegetarian and vegan diet (*p* < 0.01).

**Table 7:** Health behaviors by type and frequency of sports participation. Values display prevalences (%).

	Leisure-Time Sports		Club Sports		Sport Days/Week		
	Yes	No	Yes	No	None	1–3 Days	4–7 Days
**Daily Fruit ^1,3^**	63.9	50.7	61.4	62.8	50.3	60.5	72.4
**Daily vegetable ^1,3^**	74.2	56.6	72.1	72.3	56.9	72.3	78.9
**Fluid Intake (>2 L/day) ^1,2,3^**	28.5	15.1	34.8	23.7	15.0	23.1	41.9
Water as most common drink	76.5	73.0	76.4	76.0	73.2	75.2	79.8
**Alcohol ^1^**	82.3	75.0	82.0	81.3	75.2	83.5	79.2
**Smoking ^1,3^**	10.1	17.8	9.6	11.5	18.3	10.5	8.5

^1^ Significant difference between sports participation during leisure time (*p* < 0.05); ^2^ significant difference between club sports participation (*p* < 0.05); ^3^ significant difference between sport days/week (*p* < 0.05).

**Table 8:** Health behaviors by diet types. Values display prevalences (%).

	Mixed Diet	Vegetarian	Vegan
**Leisure-time sports participation**	88.9	88.7	83.9
**Club sports participation**	29.8	22.6	29.0
**Fluid Intake (>2 L/day)**	26.4	32.2	29.0
Water as most common drink ^1,2,3^	76.2	80.9	58.1
**Alcohol ^2^**	82.0	74.8	87.1
**Smoking**	11.3	7.8	9.7

^1^ Significant difference between mixed diet and vegetarian diet (*p* < 0.01); ^2^ significant difference between mixed diet and vegan diet (*p* < 0.01); ^3^ significant difference between vegetarian and vegan diets (*p* < 0.01).

## Data Availability

The data are not publicly available due to data protection and security laws.

## Appendix A

Results supplemental to the main text are provided within two tables, Table A1 and Table A2.

**Table A1:** Anthropometric characteristics of secondary teachers and principals by federal state and living area. Values are means ± SD and prevalence for weight categories.

	N	Age (Years)	Height (cm)	Body Weight (kg)	BMI (kg/m^2^)	Overweight/ Obesity (%)
**Burgenland**	**151**	**44.4 ± 11.4**	**170.8 + 8.1**	**69.8 ± 14.0**	**23.8 ± 3.5**	**29.8**
Urban	16	46.5 ± 9.5	169.5 + 6.1	68.9 ± 12.7	23.9 ± 3.7	31.3
Rural	135	44.1 ± 11.6	171.0 ± 8.3	69.9 ± 14.1	23.8 ± 3.5	29.6
**Carinthia**	**52**	**47.5 ± 10.7**	**170.7 ± 7.7**	**69.4 ± 12.8**	**23.7 + 3.1**	**30.8**
Urban	21	47.0 ± 10.9	171.1 ± 9.0	70.5 ± 14.3	23.9 ± 3.4	38.1
Rural	31	47.8 ± 10.7	170.3 ± 6.9	68.8 ± 11.9	23.6 ± 2.9	25.8
**Lower Austria**	**231**	**46.4 ± 11.1**	**170.1 ± 8.6**	**73.3 ± 15.6**	**25.2 ± 4.5**	**43.7**
Urban	51	48.6 ± 11.6	169.4 ± 9.0	71.3 ± 15.4	24.7 ± 3.8	39.2
Rural	180	45.8 ± 10.9	170.3 ± 8.4	73.8 + 15.6	25.4 ± 4.7	45.0
**Salzburg**	**89**	**44.3 ± 11.8**	**172.2 ± 8.6**	**71.5 ± 13.4**	**24.0 ± 3.6**	**31.5**
Urban	28	42.1 ± 12.5	169.8 ± 8.0	66.5 ± 11.9	23.0 ± 3.4	17.9
Rural	61	45.4 ± 11.4	173.3 ± 8.7	73.9 ± 13.5	24.5 ± 3.6	37.7
**Styria**	**180**	**47.3 ± 12.2**	**171.5 ± 13.6**	**71.7 ± 13.6**	**24.3 ± 3.9**	**36.7**
Urban	71	46.8 ± 12.4	171.3 ± 8.1	71.6 ± 13.7	24.3 ± 4.0	40.8
Rural	109	47.6 ±12.1	171.6 ± 8.6	71.8 ± 13.6	24.3 ± 3.9	33.9
**Tyrol**	**190**	**46.0 ± 11.6**	**171.0 + 8.5**	**70.8 ± 14.3**	**24.1 ± 3.6**	**36.8**
Urban	59	46.5 ± 12.0	171.1 ± 8.9	70.2 ± 13.6	23.8 ± 3.5	32.2
Rural	131	45.8 ± 11.5	170.9 ± 8.4	71.2 ± 14.6	24.2 ± 3.7	38.9
**Upper Austria**	**176**	**46.1 ± 10.5**	**172.1 ± 8.0**	**72.3 ± 14.6**	**24.3 ± 3.9**	**35.8**
Urban	50	45.4 + 11.4	172.8 ± 7.8	72.2 ± 11.8	24.1 ± 3.1	38.0
Rural	126	46.4 ± 10.2	171.8 ± 8.1	72.3 ± 15.6	24.3 ± 4.2	34.9
**Vienna**	**138**	**45.2 ± 12.0**	**171.2 ± 7.6**	**70.3 ± 15.6**	**23.9 ± 4.5**	**27.7**
Urban	138	45.2 ± 12.0	171.2 ± 7.6	70.3 ± 15.6	23.9 ± 4.5	27.7
Rural	None					
**Vorarlberg**	**143**	**44.7 ± 11.0**	**171.7 ± 8.9**	**70.0 ± 15.2**	**23.6 ± 3.9**	**25.9**
Urban	75	45.5 ± 10.5	171.2 ± 7.9	69.9 ± 13.2	23.8 ± 3.9	25.3
Rural	68	43.9 + 11.6	172.3 ± 10.0	70.1 ± 17.3	23.4 + 4.0	26.5

Bold—total numbers.

**Table A2:** Health behavior of secondary teachers and principals by federal state and living area. Values are in prevalences (%) and mean ± SD for days with sports.

	N	Leisure-Time Sports (%)	Club Sports (%)	Days/Week with Sport (Mean ± SD) *	Daily Fruits (%)	Daily Veggies (%)	Fluid Intake >2 L/Day (%)	Water Most Common Fluid (%)	Vegetarian /Vegan (%)	Alcohol (%)	Smoking (%)
**Burgenland**	**151**	**86.1**	**27.8**	**3.0 ± 1.3**	**58.9**	**62.9**	**25.8**	**79.5**	**12.6**	**78.1**	**6.6**
Urban	16	87.5	31.3	2.1 ± 0.9	37.5	68.8	18.8	81.3	18.8	93.8	0.0
Rural	135	85.9	27.4	3.1 ± 1.4	61.5	62.2	26.7	79.3	11.9	76.3	7.4
**Carinthia**	**52**	**88.5**	**25.0**	**3.3 ± 1.6**	**65.4**	**63.5**	**23.1**	**75.0**	**5.8**	**73.1**	**17.3**
Urban	21	85.7	19.0	3.1 ± 1.4	61.9	47.6	28.6	90.5	4.8	71.4	23.8
Rural	31	90.3	29.0	3.4 ± 1.7	67.7	74.2	19.4	64.5	6.5	74.2	12.9
**Lower Austria**	**231**	**86.6**	**24.2**	**2.8 ± 1.4**	**63.6**	**70.1**	**31.2**	**71.0**	**12.1**	**80.5**	**10.8**
Urban	51	92.2	27.5	2.9 ± 1.3	64.7	72.5	27.5	74.5	15.7	88.2	3.9
Rural	180	85.0	23.3	2.8 ± 1.5	63.3	69.4	32.2	70.0	11.1	78.3	12.7
**Salzburg**	**89**	**96.6**	**44.9**	**3.2 ± 1.6**	**68.5**	**80.7**	**34.8**	**76.4**	**13.5**	**86.5**	**9.0**
Urban	28	96.4	39.3	3.5 ± 1.7	75.0	85.7	35.7	89.3	32.1	85.7	10.7
Rural	61	96.7	47.5	3.1 ± 1.6	65.6	78.3	34.4	70.5	4.9	86.9	8.2
**Styria**	**180**	**87.2**	**26.1**	**2.8 ± 1.4**	**60.6**	**73.3**	**25.0**	**76.7**	**10.6**	**78.3**	**11.7**
Urban	71	90.1	21.1	2.7 ± 1.3	50.7	74.6	29.6	73.2	14.1	90.1	9.9
Rural	109	85.3	29.4	2.9 ± 1.4	67.0	72.5	22.0	78.9	8.3	70.6	12.8
**Tyrol**	**190**	**90.0**	**26.8**	**3.2 ± 1.5**	**68.9**	**70.0**	**28.4**	**77.9**	**7.4**	**84.7**	**7.4**
Urban	59	86.4	18.6	3.1 ± 1.3	71.2	74.6	25.4	74.6	11.9	86.4	8.5
Rural	131	91.6	30.5	3.3 ± 1.6	67.9	67.9	29.8	79.4	5.3	84.0	6.9
**Upper Austria**	**176**	**91.5**	**40.3**	**2.7 ± 1.4**	**55.1**	**74.4**	**22.7**	**75.6**	**11.9**	**85.2**	**12.5**
Urban	50	88.0	36.0	2.5 ± 1.1	58.0	76.0	16.0	70.0	14.0	88.0	14.0
Rural	126	92.9	42.1	2.8 ± 1.4	54.0	73.8	25.4	76.6	11.1	84.1	11.9
**Vienna**	**138**	**86.2**	**21.7**	**2.8 ± 1.4**	**63.8**	**78.3**	**27.5**	**76.8**	**10.1**	**76.1**	**15.9**
Urban	138	86.2	21.7	2.8 ± 1.4	63.8	78.3	27.5	76.8	10.1	76.1	15.9
Rural	None										
**Vorarlberg**	**143**	**89.5**	**30.8**	**3.1 ± 1.5**	**60.1**	**76.2**	**23.1**	**78.3**	**11.2**	**86.7**	**11.9**
Urban	75	90.7	29.3	3.0 ± 1.5	60.0	80.0	24.0	78.7	13.3	85.3	10.7
Rural	68	88.2	32.4	3.3 ± 1.5	60.3	72.1	22.1	77.9	8.8	88.2	13.2

* Of those reporting leisure-time sports.
